# Microsatellite polymorphism across the *M. tuberculosis *and *M. bovis *genomes: Implications on genome evolution and plasticity

**DOI:** 10.1186/1471-2164-7-78

**Published:** 2006-04-10

**Authors:** Vattipally B Sreenu, Pankaj Kumar, Javaregowda Nagaraju, Hampapathalu A Nagarajaram

**Affiliations:** 1 Laboratory of Computational Biology, Centre for DNA Fingerprinting and Diagnostics, ECIL Road, Nacharam, Hyderabad-76, A.P., India; 2 Laboratory of Molecular Genetics, Centre for DNA Fingerprinting and Diagnostics, ECIL Road, Nacharam, Hyderabad-76, A.P., India

## Abstract

**Background:**

Microsatellites are the tandem repeats of nucleotide motifs of size 1–6 bp observed in all known genomes. These repeats show length polymorphism characterized by either insertion or deletion (indels) of the repeat units, which in and around the coding regions affect transcription and translation of genes.

**Results:**

Systematic comparison of all the equivalent microsatellites in the coding regions of the three mycobacterial genomes, viz. *Mycobacterium tuberculosis *H37Rv, *Mycobacterium tuberculosis *CDC1551 and *Mycobacterium bovis*, revealed for the first time the presence of several polymorphic microsatellites. The coding regions affected by frame-shifts owing to microsatellite indels have undergone changes indicative of gene fission/fusion, premature termination and length variation. Interestingly, the genes affected by frame-shift mutations code for membrane proteins, transporters, PPE, PE_PGRS, cell-wall synthesis proteins and hypothetical proteins.

**Conclusion:**

This study has revealed the role of microsatellite indel mutations in imparting novel functions and a certain degree of plasticity to the mycobacterial genomes. There seems to be some correlation between microsatellite polymorphism and the variations in virulence, host-pathogen interactions mediated by surface antigen variations, and adaptation of the pathogens. Several of the polymorphic microsatellites reported in this study can be tested for their polymorphic nature by screening clinical isolates and various mycobacterial strains, for establishing correlations between microsatellite polymorphism and the phenotypic variations among these pathogens.

## Background

Microsatellites, also known as simple sequence repeats, are the short nucleotide segments comprising tandem repeating motifs of length 1–6 bp [1]. They are present in all genomes known to date [2-4], and are known to be polymorphic [5]characterized by high rates of indels of repeat units [1]. Microsatellites provide a framework for crucial genetic rearrangements with their reversible frame-shift mutations that can confer a certain degree of selective advantage on pathogenic bacteria. Microsatellite mutations are known to affect expression levels [6], switching on/off of genes [6] and even alteration of gene functions [7]. The primary cause of microsatellite polymorphism is thought to be strand slippage during DNA replication [8]. Usually errors owing to strand slippage are repaired by a three-enzyme system comprising the enzymes mutL, mutS and mutH. However, some genomes like those of the mycobacterial species lack these enzymes [9]. Hence, such genomes serve as interesting systems to investigate the rates of mutations in microsatellites and the existence of regulatory mechanisms that govern microsatellite mutations. Furthermore, these genomes present challenging and exciting systems to understand the role of microsatellite mutations in conferring genome plasticity, and in aiding the pathogens in their adaptation and evolution.

Previous reports on genomic changes in *M. tuberculosis*, were mainly concerned with single nucleotide polymorphisms (SNPs) and large-sequence polymorphisms (LSPs) (>10 bp) [10]. While the involvement of SNPs in drug resistance has been shown [11], most of the LSPs are thought to be deleterious [12]. In the present study, we show for the first time that the coding regions of the three genomes of mycobacteria (*M. tuberculosis *H37Rv [13], *M. tuberculosis *CDC1551 [10] and *M. bovis *[14]) harbor a number of polymorphic microsatellite loci associated with remarkable changes in the coding regions.

## Results and discussion

**Figure 1:**
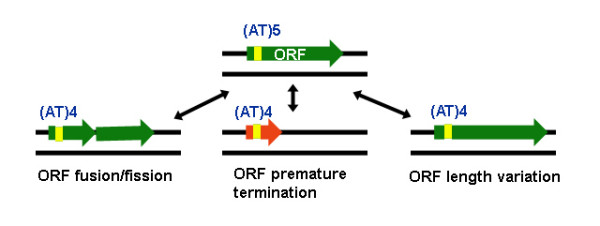
Schematic representation of the various changes observed in the coding regions (green arrows) affected by microsatellite indel mutations. In this illustration a hypothetical microsatellite tract (AT)_5 _has been shown to undergo an indel of one repeat unit causing fission/fusion, premature termination and length variation of ORFs. The bi-directional arrows (black) indicate reversible nature of the microsatellite mutations.

All the three mycobacterial genomes, *M. tuberculosis *H37Rv (MTH), *M. tuberculosis *CDC1551 (MTC) and *M. bovis *(MB) harbor about a million microsatellite tracts each, comprising of mono to hexa repeats (Sreenu, Pankaj Kumar, Nagaraju and Nagarajaram, manuscript communicated). Systematic comparison of all the equivalent microsatellites and the equivalent coding regions harboring them, in all the three genomes revealed several examples of microsatellites exhibiting length polymorphism characterized by indels of the repeat units. Frame-shifts in the coding regions owing to indels in microsatellites, were also observed. While some frame-shifts caused ORFs to split (fission) (see methods), others seemed to bring about fusion of two adjacent ORFs (with or without overlap) giving rise to a single ORF. Our study also revealed several ORFs eliminated as a result of premature termination by stop codons, and numerous other ORFs exhibiting length changes (Fig. 1). The complete list of polymorphic microsatellites along with the ORFs in which they are present is given in Table 1 (see Additional File 1 for details of the tracts, microsatellite polymorphism and outcomes). Illustrated below are some examples of microsatellites and their polymorphic effects on the coding regions.

In the MTH genome, two ORFs annotated as *gmhA *(Rv0113) and *gmhB *(Rv0114) have been identified as sedoheptulose-7-phosphate isomerase and D-α-β-D-heptose-7-biphosphate phosphatase, respectively (the TB structural genomics consortium [15]). These enzymes are known to be involved in the biosynthesis pathway of nucleotide activated glycerol-manno-heptose precursors of bacterial glycoproteins and cell surface polysaccharides [16]. Our study indicates that the ORF Rv0113 annotated as *gmhA *harbors the microsatellite (T)_4 _in MTH_,_while it is expanded to (T)_5_in the MTC genome. This expansion has resulted in a frame-shift owing to which the reading frame extends and fuses with that of the *gmhB*, thus giving rise to a fused ORF. Although it is hard to speculate the possible roles of the gmhA-gmhB fused protein in MTC, there exists a high probability of it forming a bi-functional protein with two domains.

Similarly, two adjacent ORFs viz., Rv0192A and Rv0192 in the MTH genome are observed to have fused into a single ORF (Mb0198) in the MB genome, owing to a frame-shift caused by the expansion of the microsatellite (G)_4 _to (G)_5_. Previous PhoA fusion screening studies have shown Rv0192A in MTH to act as a signal peptide [17], and in light of this it is reasonable to speculate the fused gene product in MB to be a secretory protein that may act as a surface antigen.

The ORF MT1966 in MTC encoding a functional isocitrate lyase [18], is observed to have split into two ORFs (Rv1915 and Rv1916) in MTH due to a single nucleotide deletion in the mononucleotide tract (T)_5_. The failure of these two ORFs to complement isocitrate lyase activity in MTH has been demonstrated [19]. Immunoblotting studies were unable to detect AceAa or AceAb products [18]. Subsequent studies by Betts and co-workers (2002) enabled detection of only the mRNA of AceAa, indicating the lack of expression of AceAb [20]. It is interesting to note that both the MTC and MTH genomes possess another copy of isocitrate lyase. This indicates the existence of two functional copies of the enzyme in MTC, and only a single copy in MTH. In MTC the activity of isocitrate lyase increases during the latent phase when the pathogen utilizes lipid as the energy source [21]. Redundancy in isocitrate lyase in MTC can therefore be beneficial to the pathogen, providing a greater chance of its survival in the host cell debris where lipid is used as a carbon source. However, in MTH which is cultured under laboratory conditions with no dependence on lipids as the carbon source, the duplication of the isocitrate lyase enzyme is not required. Therefore, the removal of one copy of the enzyme in MTH may not pose as a constraint for the growth of the pathogen.

On comparison, the highest number (18 ORFs) of split events is observed in the MB genome (Table 1). The expression of both parts of split genes in the MB genome, imply a favorable situation for versatile protein-protein interactions. However, it is to be noted in the cases of split ORF, the expression of the second part of the ORF is entirely dependent on the availability of regulatory signals (Shine-Dalgarno sequence) for that ORF. In the absence of a regulatory mechanism, the second part of the ORF is unexpressed. As given in Table 1, section III, the second part of all the four examples, has been annotated as psuedogene because of the absence of the Shine-Dalgarno sequence. If both the parts of the split ORFs are expressing the split subunits can act together [22,23] or in isolation resulting in different protein-protein interactions, that can be instrumental in the creation of alternate/new pathways, which in turn may eventually render greater adaptation mechanisms to the bacteria. This may well be the one of the underlying reasons for MB to have a wider host range as compared to *M. tuberculosis*.

The split ORFs encode membrane proteins, transporters, PE_PGRS, cell-wall synthesis proteins and hypothetical proteins. The membrane proteins are known to play an important role in host-pathogen interactions [24]. The majority of bacteria are thought to modify their membrane protein structures in order to escape the host immune defense system and promote colonization at various places within the host [6,24]. The PE-PGRS proteins are specific to mycobacteria and are speculated to function as surface antigens [25,26]. Truncation with respect to the second part can potentially give rise to an antigenic variant.

MTC as compared to the other genomes exhibits a greater number of cases of premature terminations (10 ORFs) (Table 1), confined to the PE_PGRS, *umaA1*, *pks5 *and some hypothetical proteins. Of these, the ORF *umaA1 *codes for a mycolic acid methyl transferase that modifies the lipids of the mycobacterial cell wall [27]. The *umaA1 *deletion mutant of MTH is observed to be more virulent than the wild-type, in the severe combined immune deficiency (SCID) mouse model [28]. However, it is difficult to categorically stress the importance of *umaA1 *in the virulence of the pathogen. This is because MTC has been shown to be less virulent in the immunocompetent mice as compared to other clinical isolates [29]. Study on an umaA1 deletion mutant of MTH in immunocompetent mice would provide clues to the role of umaA1 in virulence. In addition, it is equally possible for the other prematurely terminated ORFs to also be responsible for the less virulent nature of MTC. However, such correlations require further studies.

We also observe an appreciable number of ORFs (43 examples) in all the three genomes exhibiting length variations due to indels of repeat units in microsatellites. Many proteins in this category have been annotated as hypothetical proteins, PPE and mammalian cell entry (mce) family virulence proteins. While the length variation in some ORFs produce no effect on the function of the translated protein with the functional domains being well conserved; in others, drastic changes are observed. For example, Rv2732c in MTH as well as Mb2791c in MB code for a membrane anchoring protein of length 204aa. The equivalent ORF MT2802.1 in MTC is a shorter ORF encoding only 180aa, owing to a frame-shift caused by a single G insertion in the microsatellite tract (G)_2_. *In silico *analysis of these proteins, reveals a greater probability (0.959) of the N-terminal deleted short protein in MTC to act as a signal peptide and secrete outside, than its longer counterparts in MB and MTH that possess negligible propensities of being signal peptides and therefore for external secretion.

Although the primary focus of this communication is on microsatellite polymorphism in the coding regions, we have also examined the upstream promoter regions of the ORFs and obtained some ORFs harboring polymorphic microsatellites (data not shown). It should be noted that genes are located very close to each other in a prokaryotic genome; at times without any long intergenic region between two adjacent genes. It is probable that the coding sequence of a gene may act as a regulatory sequence for its neighboring genes. In addition to bringing about changes in the coding regions, the observed microsatellite variations may also influence regulation of regions downstream of coding sequences.

We have referred the Stanford microarray database [30], Tuberculist [31], ArrayExpress [32] and available literature on microarray analysis of mycobacterium [20,33-37] for the expression profiles of all ORFs of MTH listed in Table 1. Almost 85% of the ORFs (indicated by * in the table) display high expression profiles, including those that have undergone fission. However, further studies are necessary to verify and complement the function of these split gene products with their cognate wild-type/unsplit proteins.

It is evident from Table 1 that microsatellites with as few as two repeats display polymorphism (i.e., indels of their repeat units). This appears to contradict earlier observations of the requirement of a microsatellite length threshold for repeat expansions or contractions due to strand slippage [38,39]. Our study therefore indicates the non-dependence of strand slippage on microsatellite tract lengths. However, one should bear in mind the possibility of random mutational events leading to the observed length variation in microsatellites. For example, the genomes of *M. canetti *and *M. tuberculosis *contain the (GGGCCGC)_2 _tract in the ORF that encodes for *pks15/1*. However, the equivalent regions in the MTC and MTH genomes have a 7 bp deletion of (GGGCCGC) and in the MB genome a 6 bp deletion of (GGCCGC) [40]. Although the deletion events are independent, the resultant sequences when compared give an impression of the G tract expansion. Alternatively, it can be argued that all three genomes MB, MTC and MTH may have possessed an initial 7 bp deletion (GGGCCGC) similar to *M. canetti*, giving rise to the microsatellite tract (G)_5 _that may have subsequently expanded to (G)_6 _in MB. It is still unclear as to which of the models depict the correct picture of events for the observed microsatellite polymorphism. This is largely because of the unavailability of detailed evolutionary information of the mycobacterial pathogen. Although *M. canetti *is believed to be the root from which the other mycobacterial strains evolved, a clear understanding of the evolutionary relationship between *M. tuberculosis *and *M. bovis *is absent [41-44]. Owing to this, it is difficult to put forward precisely the path of microsatellite evolution, although several possibilities can be suggested.

The rate at which microsatellites mutate is much higher than the single-base substitutions [45,46], therefore greater variations are expected in the polymorphic loci than other regions of the genomes. Though mycobacterial genomes are enriched with microsatellite tracts (Sreenu, Pankaj, Nagaraju and Nagarajaram, manuscript communicated), surprisingly there is yet no report available on the microsatellite mediated phase variation in these bacteria. The majority of microsatellite mediated phase variations reported in pathogenic bacteria are changes in the pili [47,48], capsule [49,50] and flagella [51,52] and the mycobacteria do not possess any of these structures. According to Hallet, phase variation is "an adaptive process through which bacteria undergo frequent and reversible phenotypic changes resulting in genetic alterations in their genomes" [53]. In light of this point it is highly interesting that this work presents several polymorphic microsatellite loci that seem to have been evolutionarily 'selected' and are involved in bringing about phenotypic alterations in the coding regions namely, antigenic variation, virulence and modified host-pathogen interactions for presumably better adaptation of the pathogen.

It is tempting to speculate that some of the polymorphic microsatellites discovered in this study are those that have undergone mutations at some point of time during microbe evolution, perhaps during speciation, and thereafter remained frozen as the 'molecular fossils'. If this model is correct, then such tracts can be used as markers for species/strain identification. In any case all the loci form a good starting set to screen several isolates and strains. This would enable to study correlation between microsatellite polymorphism and the observed phenotypic variations among different isolates and strains.

An important point to be noted in connection with microsatellite polymorphism in the mycobacterial genomes is the absence of the post replicative DNA mismatch repair system mediated by *mutS, mutL *and *mutH *genes [9]. Impairment of these enzymes destabilizes mono, di and trinucleotide repeats [54]. This probably accounts for the prevalence of mono and dinucleotide microsatellite variations in mycobacterial genomes. Moreover, the absence of these enzymes appears advantageous to these pathogens, resulting in the generation of polymorphic microsatellites, thereby imparting a certain degree of plasticity to the genomes. However, the total number of microsatellites that exhibit polymorphism, and their significance in the context of pathogen adaptability, virulence and survival remains to be tested.

## Conclusion

The coding regions in the mycobacterial genomes, viz. *M. tuberculosis *H37Rv, *M. tuberculosis *CDC1551 and *M. bovis*, harbor a number of polymorphic microsatellites. The observed indel mutations in microsatellites have brought out some interesting changes in the coding regions indicative of gene fusion/fission, loss, and functional variation. From this study, it can be concluded that microsatellites form an important set of genomic elements, mutations of which are beneficial to the pathogens.

## Methods

Complete genome sequences of *M. tuberculosis *(H37Rv and CDC1551) and *M. bovis *were downloaded from the NCBI ftp site [55]. Functional annotations of the coding regions were referred to the Tuberculist website [31] and the TB structural genomics consortium site [15]. The various microsatellites in the three genomes were identified using SSRF [56]. SSRF scans a given nucleotide sequence and extracts all microsatellite tracts of motif length 1–6 bp. The extracted information includes genomic location of the tracts, repeating motifs, repeat numbers and regions (coding or non-coding or partial) in which the tracts are present. The program utilizes the GenBank annotation file "xxx.ffn" (where xxx = genome name) that has exon boundary information, using which the location of microsatellites relative to the protein coding regions is subsequently recorded. In addition the internal motif redundancy is taken care of; where a sequence of the type (AAAAGCAAAAGCAAAAGC) is represented as (AAAAGC)_3 _with the internal "A"s (AAAAGC) not considered as a separate (A)_4 _tract.

The ORFs harboring microsatellites of one genome were used as queries to search against the other two complete mycobacterial genome sequences using the BLASTN program (version 2.2.6) [57] without the repeat masking filter. The alignment hits with queried sequences comprising only indels in the microsatellites were selected for further analysis. The Tuberculist database (for H37Rv and *M. bovis*) and the NCBI (for CDC1551) were checked and confirmed to ensure that the indels in microsatellites especially those of the mononucleotide tracts were indeed authentic mutations and not the results of sequencing errors (however one can not rule out some remote possibility of sequencing artifact). Subsequently, the ORFs and their equivalent sequences were realigned using CLUSTALW [58] to reconfirm the alignment as well as the INDELS in the microsatellites. As the phylogenetic relation of these genomes is still ambiguous, a consensus of the three genomes for microsatellite categorization into premature terminations, gene fusion/fission and ORF premature termination was used.

## Authors' contributions

VBS: Computational analysis of microsatellite polymorphisms across the mycobacterial genomes and initial drafting of the manuscript

PK: Comparative analysis of functions of coding regions harbouring polymorphic microsatellites across the mycobacterial genomes

JN: Provided suggestions during the initial stages of the manuscript preparation

HAN: Project leader, project guide and in-charge of final manuscript corrections and submission

## Supplementary Material

Additional File 1 List of ORFs from *M. tuberculosis *H37Rv (MTH), *M. tuberculosis *CDC1551 (MTC) and *M. bovis *(MB) harboring polymorphic microsatellite tracts. The complete list of the polymorphic microsatellites from the mycobacterial genomes, *M.tuberculosis *H37Rv, *M.tuberculosis *CDC1551 and *M.bovis*, along with the alignments of microsatellite tracts and flanking sequences. This list provides locations of microsatellite in the genomes, microsatellite variation, details of microsatellite position in protein with respect to amino acid sequence, local sequence of the of the microsatellite tract, start and end positions of the ORF, which contains the microsatellite, coding strand information (same strand:'+', template strand:'-'), GenBank ID of a protein, function of protein and protein length Click here for file

## Figures and Tables

**Table 1:** The complete list of polymorphic microsatellites found in the coding regions of the three genomes, *M. tuberculosis *H37Rv (MTH), *M. tuberculosis *CDC1551 (MTC) and *M. bovis *(MB). Please note that the microsatellites in the intergenic regions are not reported here. The table lists the ORFs (given by their gene id) harboring the polymorphic microsatellites. The first column denotes microsatellite tract and its observed mutation in the form of insertion/deletion of repeat units leading to expansion or contraction of the microsatellite. As discussed in the text evolutionary relationship among the three genomes, is not established clearly. Therefore, we have followed a consensus approach where the observed event being a case of insertion or deletion of a repeat, is decided by the number of genomes in which the repeat number is conserved (given in bold text). For example, G4↔5 denotes that two of the genomes possess the tract G4 while in the third genome it exists as G5, and therefore it is regarded as an event of insertion leading to microsatellite expansion. Accordingly, the effect (fusion/fission, premature termination, length variation) on the coding region is also displayed.

**Mutation**	**Function**	**MTH**	**MTC**	**MB**
**I Mutation leading to ORF splitting (33)**				

**a) Fusion with overlaping orf (5)**				
**G4**↔5	Membrane protein	*Rv0192A (100), Rv0192 (366)	**MT0202 (366), 2**^nd^**might be 100aa #**	Mb0198 (352)
**G4**↔5	Membrane protein mce2b	Rv0590A (84), *Rv0590 (275)	**MT0619 (287), 2**^nd^**might be 84 aa #**	Mb0605 (343)
**G7**↔11	FrdB, frdC	*Rv1553 (247), *Rv1554 (126)	**MT1604 (247), MT1605 (126)**	Mb1579 (374)
**T4**↔5	Hypothetical protein	*Rv3338 (214) *Rv3337 (128)	**MT3441 (248)**	Mb3370 (297)
**T2**↔3	Cut5a, cut5b truncated cutinase	Rv3724A (80), *Rv3724B(187)	**MT3827 (207)**	**Mb3751 (233)**
**b) Fusion with non-overlaping orf (4)**				
**T5**↔4	GmhA	*Rv0113 (196), *Rv0114 (190)	MT0122 (420)	**Mb0117 (196) Mb0118 (190)**
**G5**↔6	Pks1, pks15 polyketide synthase	*Rv2946c (1616), *Rv2947c(496)	**MT3018 (1620), MT3021.1 (496)**	Mb2971c (2112)
**CG3**↔2	Hypothetical protein	*Rv2974c (470), *Rv2975c (84)	**MT3052 (470), MT3052.1(92)**	Mb2999c (553)
**T2**↔3	*dTDP-glucose 4,6-dehydratase	Rv3784 (326), *Rv3785 (357)	MT3893 (712)	**Mb3813 (326), Mb3814 (357)**
**c) Fission with overlaping orf (8)**				
**G4**↔**5**	Flavo protein, electron acceptor,	Rv2251A (139) *Rv2251(475)	**MT2311 (529)**	**Mb2275 (529)**
**G5**↔6	Conserved hypothetical protein	*Rv2879c (189) Rv2880c (275)	**MT2947 (364)**	**Mb2904c (364)**
**G4**↔3	Conserved hypothetical protein	*Rv0740 (175)	MT0765 (82), MT0766 (120)	**31791926 (175)**
**G3**↔2	fusA2	*Rv0120c (714)	**MT0128 (714)**	Mb0124c (597), Mb0125c (117)
**G2↔**3	*Pks6	**Rv0405 (1402)**	**MT0418 (1402)**	Mb0412 (460), Mb0413 (946)
**T6**↔7	PstB	*Rv0933 (276)	**MT0960 (276)**	Mb0957 (71), Mb0958 (213)
**C2↔**3	*drug transporter	**Rv1877 (687)**	**MT1926 (687)**	Mb1908 (511), Mb1909 (404)
**C6↔5**	LppO	*Rv2290 (171)	**MT2347 (192)**	Mb2312 (51), Mb2313 (121)
**d) Fission with non-overlaping orf (16)**				
**T5**↔4	aceAa, aceAb	*Rv1915 (367 *Rv1916 (398)	**MT1966 (766)**	**Mb1950 (766)**
**G5**↔6	Conserved hypothetical protein	*Rv2561 (97) *Rv2562 (129)	**MT2638 (212)**	**Mb2591 (245)**
**T2↔**3	Conserved transmembrane protein	Rv3453 (110), *Rv3454 (422)	**MT3561 (562)**	**Mb3483 (561)**
**C5↔**4	mmpL1	*Rv0402c (958)	**MT0412 (958)**	Mb0408c (367), Mb0409c (591)
**C7**↔6	Hypothetical	*Rv0698 (203)	**Might be 203 aa #**	Mb0717 (109), Mb0718 (77)
**T3**↔2	3-ketosteroid-delta-1-dehydrogenase	*Rv0785 (566)	**MT0809 (566)**	Mb0807 (191), Mb0808 (368)
**T2**↔3	cobL	**Rv2072c (390)**	**MT2132 (390)**	Mb2098c (294), Mb2099c (62)
**TG2**↔1	*Probable transposase	**Rv2424c (333)**	**MT2497 (333)**	Mb2447c (230), Mb2448c (97)
**G7**↔15	PE_PGRS	*Rv2490c (1660)	**MT2564 (1665)**	Mb2517c (1150), Mb2518c (509)
**GC3**↔2	transglutaminase family protein	**Rv2566 (1140)**	**MT2642 (1156)**	Mb2595 (533), Mb2596 (597)
**T4↔**3	ugpA	*Rv2835c (303)	**MT2901 (303)**	Mb2859c (180), Mb2860c (123)
**C2**↔3	fadE22	*Rv3061c (721)	**MT3147 (721)**	Mb3087c (600), Mb3088c (114)
**C4**↔3	mesT	*Rv3176c (318)	**MT3265 (339)**	Mb3201c (105), Mb3202c (208)
**C4**↔3	Cyp142	**Rv3518c (398)**	**MT3619 (372)**	Mb3547c (193), Mb3548c (205)
**A6**↔7	hypothetical protein	*Rv3773c (194)	**MT3882 (194)**	Mb3801c (114), Mb3802c (78)
**C2↔**3	conserved membrane protein	*Rv3894c (1396)	**MT4010 (1396)**	Mb3923c (561), b3924c (833)
**II) Muation leading to premature termination (13)**				
**C7**↔8	Oxido-reductase	*Rv0161 (449)	MT0170 (pt)	**Mb0166 (449)**
**T5**↔4	umaA1	*Rv0469 (286)	MT0485 (pt)	**Mb0478 (286)**
**G4**↔3	Cysteine synthase	*Rv0848 (372)	MT0871 (pt)	**Mb0871 (372)**
**G3↔**2	Membrane transport	*Rv0849 (419)	MT0872 (pt)	**Mb0872 (419)**
**A2**↔3	Hypothetical protein	-	**MT1025.1 (46)**	-
**G4**↔3	polyketide synthase pks5	*Rv1527c (2108)	Prematurely terminated	**Mb1554c (2108)**
**G3**↔2	Conserved hypothetical	*Rv1533 (375)	Prematurely terminated	**31792719 (375)**
**G7↔**8	*PE_PGRS(wag22) Antigen	Rv1759c (914)	**MT1807 (pt) #**	**Mb1789c (820), Mb1790c (94)**
**G3**↔2	PE_PGRS	**Rv2126c (256)**	MT2185 (pt)	**Mb2150c (256)**
**G2**↔3	Hypothetical protein	**Not annotated as orf #**	**MT2401.2 (69)**	Prematurely terminated
**CGCGC2**↔3	Oxidoreductase	*Rv3093c (334)	MT3177 (pt)	**Mb3120c (334)**
**A3**↔2	*Conserved hypothetical protein	Prematurely terminated	MT3855 (314) *	**Not annotated as orf #**
**G3**↔2	MycP2, membrane-anchored serine protease	**Rv3886c (550)**	MT4001 (pt)	**Mb3916c (550)**
**III) Mutation leading to ORF splitting and 2**^nd^**splitted part is annotated as psuedogene (4)**				
**C7↔**8	Glycolipid sulfotransferase	*Rv1373 (326)	**MT1418 (320)**	Mb1407 (265) 2^nd ^part is prematurely terminated
**C6↔**5	Hypothetical	*Rv1718 (272)	**MT1757 (386) #**	Mb1746 (207) Mb1747 (pt)
**G7↔**8	GlpK glycerol kinase	*Rv3696c (517)	**MT3798 (517)**	Mb3721c (pt) Mb3722c (251)
**C2↔**3	sigM	*Rv3911 (222)	**MT4030 (196)**	**Mb3941 (196), Mb3942(pt)**
**IV) Mutation leading to length variation of orf (43)**				
**a) Length increase from C-terminal (11)**				
**T5↔**4	CtpI	*Rv0107c(1632)	**MT0116 (1625)**	**Mb0111c (1625)**
**G4**↔3	Hypothetical protein	*Rv0607 (128)	MT0636 (147)	**Mb0623 (128)**
**G3**↔2	*lldD1*	*Rv0694 (396)	MT0721 (419)	**Mb0713 (396)**
**C4**↔5	*NusB*	*Rv2533c(156)	MT2608 (290)	**Mb2562c (156 extra aa)**
**G3↔**2	transport proteins	*Rv3239c (1048)	MT3337(1065)	**Mb3267c (1048)**
**GC4**↔5	Hypothetical protein	*Rv0739 (268)	**MT0764 (268)**	Mb0760 (282)
**A3**↔2	Hypothetical protein	*Rv1046c (174)	**MT1075.1 (262)**	**Mb1075c (197)**
**C5**↔4	Conserved hypothetical protein	**Rv1760 (502)**	**MT1809 (531)**	Mb1791 (509)
**GC2**↔1	hflX	*Rv2725c (495)	MT2797 (556)	**Mb2744c (495)**
**T4↔3**	integral membrane	*Rv3162c (145)	**MT3251 (145)**	Mb3187c (196)
**C3↔2**	ESAT-6 like protein	*Rv3890c (95)	**MT4005 (95)**	Mb3919c (124)
**b) Length increase from N-terminal (3)**				
**G3**↔2	Conserved hypothetical protein	*Rv1246c (97)	MT1284 (143)	**Mb1278c (97)**
**AC5↔**6	lprJ	*Rv1690 (127) S prob 0.939	**MT1729 (127) S prob 0.939**	Mb1716 (139) S prob 0.005
**G5↔**4	Conserve membrane protein	*Rv3693 (440) S prob: 0.994	MT3795 (475) S prob: 0.0	**Mb3718 (440)**
**c) Length decrease from N-terminal (6)**				
**G2**↔3	PBP-4 (penicilline binding)	*Rv0907(532)	MT0930 (562)	**Mb0931 (516)**
**G6**↔5	moac2	*Rv0864 (167)	**MT0887 (167)**	Mb0888 (142)
**T3↔**2	Membrane protein	*Rv1101c (385) s prob 0.708	**MT1133 (385) s prob: 0.708**	Mb1131c (342) s prob 1
**A6**↔5	aroE	*Rv2552c (269)	**MT2629 (269)**	Mb2582c (260)
**G2↔**3	Memrane protein	**Rv2732c (204)**	MT2802.1(180) S prob: 0.959.	**Mb2791c (204) **S prob 0.000
**G3↔**2	Conserve membrane protein	*Rv3885c(537) S prob: 0.993	MT4000 (422) S prob: 0.0	**Mb3915c (537)**
**d) Length decrease from C-terminal (12)**				
**G6**↔5	membrane protein	*Rv0010c (141)	**MT0013 (141)**	Mb0010c (111)
**A3**↔2	Conserved hypothetical protein	**Rv0025 (120)**	MT0028 (90)	**Mb0026 (120)**
**C3↔**2	NLP/P60 Antigen	*Rv0024 (281)	**MT0027 (281)**	Mb0024 (277)
**C7↔**8	mce2D	*Rv0592 (508)	**MT0622 (508)**	Mb0607 *(478)*
**A8**↔7	PPE	*Rv0878c (443)	**MT0901 (444)**	Mb0902 (438)
**C5**↔6	PPE	*Rv1168c (346)	**MT1205 (346)**	Mb1201c (180)
**CG5**↔4	Secretory protein	*Rv1312 (147)	MT1352 **(147)**	Mb1344 (144)
**G4**↔3	Hypothetical protein	*Rv1725c (236)	MT1766 (187)	**Mb1754c (236)**
**TG2**↔1	SseB	**Rv2291 (284)**	**MT2348 (268)**	Mb2314 (256)
**G2**↔3	UDP-glucosyltransferases	*Rv2958c (428)	**MT3034 (428)**	Mb2982c (366)
**G3**↔2	Cyclase	*Rv3377c (501)	**MT3487 (501)**	Mb3411c (483)
**G2**↔3	Conserve hypothetical	*Rv3836 (137)	**MT3944 (133)**	Mb3886 (116)
**V) Inframe mutation (11)**				
**CGGCCC1**↔2	Lipoprotein, s, lipid attach	*Rv0838 (256)	**MT0860 (231)**	Mb0861 (258)
**GGC5**↔4	PE-PGRS	*Rv0872c (606)	**MT0894 (609)**	**Mb0896c (608)**
**CGG5**↔4	PPE	*Rv2356c (615)	**MT2425 (615)**	Mb2377c (614)
**GCC4**↔3	PE_PGRS	**Rv2396 (361)**	**MT2467.1 (382)**	Mb2418 (360)
**TCGACG1**↔2	Hypothetical protein	*Rv1434 (45)	**MT1478 (47)**	**Mb1469 (45)**
**G8↔**11	membrane protein	*Rv2081c (146)	**MT2143 (150)**	Mb2107c (147)
**GGC4**↔3	Gdh	*Rv2476c (1624)	**MT2551 (1624)**	Mb2503c (1623)
**G6**↔3	Transcription regulatory	*Rv2621c (224)	**MT2696 (224)**	Mb2654c (223)
**GCG5**↔4	PPE	*Rv3159c (590)	**MT3247 (603)**	Mb3183c (589)
**TGG4↔**5	Memrane protein	**Rv2799 (209)**	**MT2867.1 (209)**	Mb2822 (210)
**CCG4↔**3	moeZ	*Rv3206c (392)	**MT3301 (392)**	Mb3231c (391)

Sp: Signal peptide probability (predicted using SignalP [59])

Pt: Prematurely terminated

**Red: **Membrane proteins (predicted using THHMM [60])

**Green: **Second part becomes pseudo gene because of absence of Shine-Dalgarno sequence

**Blue**: Known antigens (from Tuberculist [31])

* Expression of ORFs of *M. tuberculosis *H37Rv known from (Tuberculist [31], Stanford microarray database [30], ArrayExpress [32]) and from references [33-37]. In some entries in column 2, the * mark denotes information on known expression from different literature but not from microarray data. The expression profile data of MTC and MB are not available on the public domain databases and therefore not given in this table.

# Mutation is absent and also the region has not been annotated as ORF
