# Supplementary material for: Microsatellite polymorphism across the M. tuberculosis and M. bovis genomes: Implications on genome evolution and plasticity
Source: BMC Genomics. 2006 Apr 10;7:78. doi: 10.1186/1471-2164-7-78 (PMC1501019; doi:10.1186/1471-2164-7-78)
Supplement: Additional File 1 — List of ORFs from M. tuberculosis H37Rv (MTH), M. tuberculosis CDC1551 (MTC) and M. bovis (MB) harboring polymorphic microsatellite tracts. The complete list of the polymorphic microsatellites from the mycobacterial genomes, M.tuberculosis H37Rv, M.tuberculosis CDC1551 and M.bovis, along with the alignments of microsatellite tracts and flanking sequences. This list provides locations of microsatellite in the genomes, microsatellite variation, details of microsatellite position in protein with respect to amino acid sequence, local sequence of the of the microsatellite tract, start and end positions of the ORF, which contains the microsatellite, coding strand information (same strand:'+', template strand:'-'), GenBank ID of a protein, function of protein and protein length [file 1471-2164-7-78-S1.doc]

List of ORFs from *M. tuberculosis* H37Rv (MTH), *M. tuberculosis* CDC1551 (MTC) and *M. bovis* (MB) harboring polymorphic microsatellite tracts.

First row gives the information of location of microsatellite in the genomes, microsatellite variation

Second third and fourth rows give the details of microsatellite position in protein w.r.t amino acid sequence, local sequence of the of the microsatellite tract, start and end positions of the ORF, which

contains the microsatellite, coding strand information (same strand:’+‘, template strand:’-‘),

GenBank ID of a protein, function of protein and protein length

MTH:223750 MTC:223864 MB:223944 g4↔5

63 ccggaggtcccgggg-ccccgggggcacag 223562 224662 + Rv0192 hypothetical protein 366

63 ccggaggtcccgggg-ccccgggggcacag 223676 224776 + MT0202 conserved hypothetical protein 366

48 ccggaggtcccgggggccccgggggcacag 223799 224857 + Mb0198 PROBABLE MEMBRANE PROTEIN 352

MTH:688791 MTC:690242 MB:690036 g4↔5

254 cctagccgacctgct-ggggcggatcgtcc 688030 688857 + Rv0590 hypothetical protein Rv0590 275

266 cctagccgacctgct-ggggcggatcgtcc 689445 690308 + MT0619 virulence factor mce family protein 287

254 cctagccgacctgctgggggcggatcgtcc 689275 690306 + Mb0605 MCE-FAMILY PROTEIN MCE2B 343

MTH:1760163 MTC:1760319 MB:1744180 g7↔11

243 agttgctgttccctt----gggggggcgga 1759433 1760176 + Rv1553 frdB 247

243 agttgctgttccctt----gggggggcgga 1759589 1760332 + MT1604 fumarate reductase, iron-sulfur protein 247

243 agttgctgttcccttgggggggggggcgga 1743450 1744574 + Mb1579 PROBABLE FUMARATE REDUCTASE [IRON-SULFUR SUBUNIT] FRDB and [MEMBRANE ANCHOR SUBUNIT] FRDC (FUMARATE DEHYDROGENASE) (FUMARIC HYDROGENASE) 374

MTH:3723902 MTC:3721514 MB:3681155 t4↔5

1 cgggcacccggtttt-gtcctcggcggtgc 3723900 3724544 + Rv3338 hypothetical protein 214

35 cgggcacccggtttt-gtcctcggcggtgc 3721410 3722156 + MT3441 hypothetical protein 248

83 cgggcacccggtttttgtcctcggcggtgc 3680905 3681798 + Mb3370 CONSERVED HYPOTHETICAL PROTEIN 297

MTH:4169666 MTC:4161982 MB:4107129 t3↔2

21 tcgacgcactgcgtttcccaggttggcgcc 4169603 4170166 + Rv3724 cut5 protein 187

41 tcgacgcactgcgtttcccaggttggcgcc 4161859 4162482 + MT3827 serine esterase, cutinase family 207

67 tcgacgcactgcgtt-cccaggttggcgcc 4106927 4107628 + Mb3751 PROBABLE CUTINASE PRECURSOR CUT5 233

MTH:137853 MTC:137847 MB:137893 t5↔4

179 atctcacatcgtttttattcatgcgatctc 137317 137907 + Rv0113 gmhA 196

202 atctcacatcgtttt-attcatgcgatctc 137242 138504 + MT0122 phosphoheptose isomerase 420

179 atctcacatcgtttttattcatgcgatctc 137357 137947 + Mb0117 PROBABLE PHOSPHOHEPTOSE ISOMERASE GMHA 196

MTH:3296374 MTC:3290698 MB:3253066 g5↔6

488 cgaaagcaccggggg-ccgcggccgtcgat 3296347 3297837 - Rv2947c pks15 496

488 cgaaagcaccggggg-ccgcggccgtcgat 3290671 3292161 - MT3021.1 polyketide synthase 496

487 cgaaagcaccggggggccgcggccgtcgat 3248190 3254528 - Mb2971c PROBABLE POLYKETIDE SYNTHASE PKS1 2112

MTH:3331360 MTC:3325683 MB:3288051 cg3↔2

83 ctcgcggccggcgcgcgttgaacggagctc 3331355 3331609 - Rv2975c hypothetical protein 84

91 ctcgcggccggcgcgcgttgaacggagctc 3325678 3325956 - MT3052.1 hypothetical protein 92

83 ctcgcggccggcgcg--ttgaacggagctc 3286638 3288299 - Mb2999c CONSERVED HYPOTHETICAL ALANINE RICH PROTEIN 553

MTH:4231228 MTC:4223544 MB:4167526 t2↔3

325 agcggttccagcgtt-ctctaatacacccg 4230253 4231233 + Rv3784 epiB 326

325 agcggttccagcgtttctctaatacacccg 4222569 4224707 + MT3893 NAD-dependent epimerase/dehydratase family protein 712

325 agcggttccagcgtt-ctctaatacacccg 4166551 4167531 + Mb3813 POSSIBLE DTDP-GLUCOSE 4,6-DEHYDRATASE RFBB 326

MTH:2525725 MTC:2522988 MB:2504790 g4↔5

54 gcggccccaacgggggaggacgccgtcgcc 2525563 2526990 + Rv2251 hypothetical protein 475

108 gcggccccaacgggg-aggacgccgtcgcc 2522663 2524252 + MT2311 alkyl-dihydroxyacetonephosphate synthase, putative 529

108 gcggccccaacgggg-aggacgccgtcgcc 2504465 2506054 + Mb2275 POSSIBLE FLAVOPROTEIN 529

MTH:3190149 MTC:3184305 MB:3146666 g5↔6

175 ggtgttcatgggtatggggggagccgctgg 3189848 3190675 - Rv2880c hypothetical protein 275

175 ggtgttcatgggtat-gggggagccgctgg 3183738 3184832 - MT2947 conserved hypothetical protein 364

175 ggtgttcatgggtat-gggggagccgctgg 3146099 3147193 - Mb2904c CONSERVED HYPOTHETICAL PROTEIN 364

MTH:831930 MTC:834122 MB:833756 g4↔3

52 cgtgaggccgaaggggttcgggacatgatg 831774 832301 + Rv0740 hypothetical protein Rv0740 175

79 cgtgaggccgaaggg-ttcgggacatgatg 833885 834133 + MT0765 hypothetical protein 82

52 cgtgaggccgaaggggttcgggacatgatg 833600 834127 + Mb0761 CONSERVED HYPOTHETICAL PROTEIN 175

MTH:147452 MTC:147445 MB:147492 g3↔2

106 gtgggtgagctgcgggccgggttgcgggcc 145625 147769 - Rv0120c fusA2 714

106 gtgggtgagctgcgggccgggttgcgggcc 145618 147762 - MT0128 translation elongation factor G 714

106 gtgggtgagctgtgg-ccgggttgcgggcc 147457 147810 - Mb0125c PROBABLE ELONGATION FACTOR G FUSA2A [FIRST PART] (EF-G) 117

MTH:487098 MTC:488547 MB:488116 g2↔3

456 cggattgatcaaggc-ggtgttgatggttg 485729 489937 + Rv0405 pks6 1402

456 cggattgatcaaggc-ggtgttgatggttg 487180 491388 + MT0418 polyketide synthase 1402

456 cggattgatcaaggcgggtgttgatggttg 486749 488131 + Mb0412 PROBABLE MEMBRANE BOUND POLYKETIDE SYNTHASE PKS6A [FIRST PART] 460

MTH:1041453 MTC:1041468 MB:1041921 t6↔7

64 taagacgactttttt-gcgcaccctaaacc 1041262 1042092 + Rv0933 pstB 276

64 taagacgactttttt-gcgcaccctaaacc 1041277 1042107 + MT0960 phosphate ABC transporter, ATP-binding protein 276

64 taagacgactttttttgcgcaccctaaacc 1041730 1041945 + Mb0957 PHOSPHATE-TRANSPORT PROTEIN ABC TRANSPORTER PSTBa [FIRST PART] 71

MTH:2126703 MTC:2123926 MB:2116914 c2↔3

267 ctggagggccgcgcc-gctgcggccatcct 2125902 2127965 + Rv1877 hypothetical protein 687

267 ctggagggccgcgcc-gctgcggccatcct 2123125 2125188 + MT1926 drug transporter 687

267 ctggagggccgcgcccgctgcggccatcct 2116113 2117648 + Mb1908 PROBABLE CONSERVED INTEGRAL MEMBRANE PROTEIN [FIRST PART] 511

MTH:2562713 MTC:2558561 MB:2540311 c6↔5

39 gccgggttctccccccagtgcgccgtcagc 2562597 2563112 + Rv2290 lppO 171

60 gccgggttctccccccagtgcgccgtcagc 2558382 2558960 + MT2347 lipoprotein, putative 192

39 gccgggttctccccc-agtgcgccgtcagc 2540195 2540350 + Mb2312 Probable conserved lipoprotein lppOa 51

MTH:2161346 MTC:2158639 MB:2151628 t5↔4

295 atggtgcggcgtttt-acgaactgggcgtc 2160461 2161564 + Rv1915 aceAa 367

295 atggtgcggcgtttttacgaactgggcgtc 2157754 2160054 + MT1966 isocitrate lyase 766

295 atggtgcggcgtttttacgaactgggcgtc 2150743 2153043 + Mb1950 PROBABLE ISOCITRATE LYASE aceA (ISOCITRASE) (ISOCITRATASE) (ICL) 766

MTH:2881601 MTC:2877748 MB:2849055 g5↔6

65 ttgggcaccaggggggcaacaccagtgtcc 2881407 2881700 + Rv2561 hypothetical protein 97

32 ttgggcaccaggggg-caacaccagtgtcc 2877653 2878291 + MT2638 hypothetical protein 212

65 ttgggcaccaggggg-caacaccagtgtcc 2848861 2849598 + Mb2591 CONSERVED HYPOTHETICAL PROTEIN 245

MTH:3874721 MTC:3860674 MB:3822426 t2↔3

107 gacctggatctgatttacccgcggtagcgg 3874400 3874732 + Rv3453 hypothetical protein 110

108 gacctggatctgatt-acccgcggtagcgg 3860350 3862038 + MT3561 hypothetical protein 562

107 gacctggatctgatt-acccgcggtagcgg 3822105 3823790 + Mb3483 PROBABLE CONSERVED TRANSMEMBRANE PROTEIN 561

MTH:481530 MTC:481623 MB:482549 c5↔4

566 cgacgacttctggcgccccctcagaagttt 480353 483229 - Rv0402c mmpL1 958

566 cgacgacttctggcgccccctcagaagttt 480446 483322 - MT0412 membrane protein, MmpL family 958

567 cgacgacttctggcg-cccctcagaagttt 482474 484249 - Mb0409c PROBABLE CONSERVED TRANSMEMBRANE TRANSPORT PROTEIN MMPL1A [FIRST PART] 591

MTH:796455 MTC:801110 MB:800906 c7↔6

101 acaacgacacgccgaccccccctacaccca 798831 799442 + Rv0698 hypothetical protein Rv0698 203

acaacgacacgccgaccccccctacaccca 800806 801417 THIS REGION IS NOT ANNOTATED AS AN ORF

101 acaacgacacgccga-cccccctacaccca 800602 800931 + Mb0717 CONSERVED HYPOTHETICAL PROTEIN [FIRST PART] 109

MTH:879739 MTC:881883 MB:881932 t3↔2

134 cgggctgaagatctttccgctggtgggctg 879338 881038 + Rv0785 hypothetical protein 566

134 cgggctgaagatctttccgctggtgggctg 881482 883182 + MT0809 3-ketosteroid-delta-1-dehydrogenase, putative 566

134 cgggctgaagatctt-ccgctggtgggctg 881531 882106 + Mb0807 CONSERVED HYPOTHETICAL PROTEIN [FIRST PART] 191

MTH:2329856 MTC:2332191 MB:2313774 t2↔3

96 gacaacgtgaccgtg-ttgccgcacgtgtc 2328972 2330144 - Rv2072c cobL 390

96 gacaacgtgaccgtg-ttgccgcacgtgtc 2331307 2332479 - MT2132 precorrin-6y c5,15-methyltransferase 390

43 gacaacgtgaccgtgtttgccgcacgtgtc 2313714 2313902 - Mb2099c Probable precorrin-6y methyltransferase CobLa [FIRST PART]

62

MTH:2721574 MTC:2718426 MB:2689758 tg2↔1

67 agcgcctgggcaatgtgcttcaagacgcgg 2720774 2721775 - Rv2424c hypothetical protein 333

67 agcgcctgggcaatgtgcttcaagacgccg 2717626 2718627 - MT2497 IS1558, transposase 333

67 agcgcctgggcaatg--cttcaagacgccg 2689667 2689960 - Mb2448c PROBABLE TRANSPOSASE [FIRST PART] 97

MTH:2804941 MTC:2800431 MB:2771739 g7↔15

431 gcggagccggggggg--------cgtcggg 2801252 2806234 - Rv2490c PE_PGRS 1660

436 gcggagccggggggg--------cgtcggg 2796742 2801739 - MT2564 PE_PGRS family protein 1665

431 gcggagccgggggggggggggggcgtcggg 2771504 2773033 - Mb2518c PE-PGRS FAMILY PROTEIN [FIRST PART] 509

MTH:2887965 MTC:2884111 MB:2855418 gc3↔2

531 ccgcgctgctggcgcgcctcgatgaggcca 2886371 2889793 + Rv2566 hypothetical protein 1140

547 ccgcgctgctggcgcgcctcgatgaggcca 2882469 2885939 + MT2642 transglutaminase family protein 1156

531 ccgcgctgctggcgc--ctcgatgaggcca 2853824 2855425 + Mb2595 LONG CONSERVED HYPOTHETICAL PROTEIN [FIRST PART] 533

MTH:3141964 MTC:3136181 MB:3098540 t4↔3

85 tgttcaacacggcggttttcaccggtgccg 3141308 3142219 - Rv2835c ugpA 303

85 tgttcaacacggcggttttcaccggtgccg 3135523 3136434 - MT2901 sugar ABC transporter, permease protein 303

84 tgttcaacacggcgg-tttcaccggtgccg 3098421 3098792 - Mb2860c PROBABLE Sn-GLYCEROL-3-PHOSPHATE TRANSPORT INTEGRAL MEMBRANE PROTEIN ABC TRANSPORTER UGPAA [FIRST PART] 123

MTH:3425093 MTC:3420887 MB:3381642 c2↔3

110 ggcacggctgttgcc-ggcgctgattgacg 3423259 3425424 - Rv3061c fadE22 721

110 ggcacggctgttgcc-ggcgctgattgacg 3419053 3421218 - MT3147 acyl-CoA dehydrogenase, putative 721

111 ggcacggctgttgcccggcgctgattgacg 3381630 3381974 - Mb3088c PROBABLE ACYL-COA DEHYDROGENASE FADE22a [FIRST PART] 114

MTH:3544699 MTC:3542336 MB:3501951 c4↔3

199 cgccccattcttgcgccccgtcttctcgcg 3544341 3545297 - Rv3176c lipS 318

220 cgccccgttcttgcgccccgtcttctcgcg 3541978 3542997 - MT3265 conserved hypothetical protein 339

200 cgccccattcttgcg-cccgtcttctcgcg 3501924 3502550 - Mb3202c PROBABLE EPOXIDE HYDROLASE MESTA [FIRST PART] (EPOXIDE HYDRATASE) (ARENE-OXIDE HYDRATASE) 208

MTH:3954928 MTC:3947715 MB:3898146 c4↔3

196 acggcgagcggaccccaccgacgacctggt 3954321 3955517 - Rv3518c hypothetical protein 398

170 acggcgagcggaccccaccgacgacctggt 3947108 3948226 - MT3619 P450 heme-thiolate protein 372

197 acggcgagcggaccc-accgacgacctggt 3898119 3898736 - Mb3548c PROBABLE CYTOCHROME P450 MONOOXYGENASE 142 CYP142A [FIRST PART] 205

MTH:4218667 MTC:4210983 MB:4154964 a6↔7

52 tacgacgtgaaaaaa-ctaaccgagcattt 4218238 4218822 - Rv3773c hypothetical protein 194

52 tacgacgtgaaaaaa-ctaaccgagcattt 4210554 4211138 - MT3882 conserved hypothetical protein 194

52 tacgacgtgaaaaaaactaaccgagcattt 4154884 4155120 - Mb3802c CONSERVED HYPOTHETICAL PROTEIN [FIRST PART] 78

MTH:4378139 MTC:4370444 MB:4312099 c2↔3

770 accaaccgctggatg-ccgccgacgccgag 4376259 4380449 - Rv3894c hypothetical protein 1396

770 accaaccgctggatg-ccgccgacgccgag 4368564 4372754 - MT4010 FtsK/SpoIIIE family protein 1396

770 accaaccgctggatgcccgccgacgccgag 4311909 4314410 - Mb3924c POSSIBLE CONSERVED MEMBRANE PROTEIN [FIRST PART] 833

MTH:191390 MTC:191560 MB:191582 c7↔8

262 acatcttggcgttcg-cccccccgtcgagg 190605 191954 + Rv0161 hypothetical protein Rv0161 449

acatcttggcgttcgccccccccgtcgagg 190775 192125 Non-coding

262 acatcttggcgttcg-cccccccgtcgagg 190797 192146 + Mb0166 PUTATIVE OXIDOREDUCTASE 449

MTH:559950 MTC:561401 MB:560971 t5↔4

21 tttccgacgagtttttctcactgtttctag 559886 560746 + Rv0469 umaA1 286

tttccgacgagtttt-ctcactgtttctag 561337 562196 Non-coding

21 tttccgacgagtttttctcactgtttctag 560907 561767 + Mb0478 POSSIBLE MYCOLIC ACID SYNTHASE UMAA1 286

MTH:945803 MTC:945705 MB:946560 g4↔3

289 ggcggctggagcgtcggggcggtcgccctg 944936 946054 + Rv0848 cysM3 372

ggcggctggagcgtc-gggcggtcgccctg 944838 945955 Non-coding

289 ggcggctggagcgtcggggcggtcgccctg 945693 946811 + Mb0871 POSSIBLE CYSTEINE SYNTHASE A CYSK2 (O-ACETYLSERINE SULFHYDRYLASE) (O-ACETYLSERINE (THIOL)-LYASE) (CSASE) 372

MTH:946843 MTC:946744 MB:947600 g3↔2

263 ctggtcgccgtcggcgggcagctgcgcatc 946054 947313 + Rv0849 hypothetical protein Rv0849 419

ctggtcgccgtcggc-ggcagctgcgcatc 945955 947213 Non-coding

263 ctggtcgccgtcggcgggcagctgcgcatc 946811 948070 + Mb0872 PROBABLE CONSERVED INTEGRAL MEMBRANE TRANSPORT PROTEIN 419

MTH:1113736 MTC:1113777 MB:1114189 a2↔3

ccttgtggtaccggtaaagtgctgtggcga 1113632 1113773 Non-coding

35 ccttgtggtaccggt-aagtgctgtggcga 1113673 1113813 + MT1025.1 hypothetical protein 46

ccttgtggtaccggtaaagtgctgtggcga 1114085 1114226 Non-coding

MTH:1725177 MTC:1725332 MB:1709192 g4↔3

1077 gcgtacatcgccgaaggggccggcgacacg 1722081 1728407 - Rv1527c pks5 2108

gcgtacatcgccgaa-gggccggcgacacg 1722238 1728563 Non-coding

1077 gcgtacatcgccgaaggggccggcgacacg 1706096 1712422 - Mb1554c Probable polyketide synthase pks5 2108

MTH:1733886 MTC:1734042 MB:1717901 g3↔2

93 gctcatacccgaagggcatcgcaccttcgt 1733608 1734735 + Rv1533 hypothetical protein 375

gctcatacccgaagg-catcgcaccttcgt 1733764 1734890 Non-coding

93 gctcatacccgaagggcatcgcaccttcgt 1717623 1718750 + Mb1560 CONSERVED HYPOTHETICAL PROTEIN 375

MTH:1992323 MTC:1990145 MB:1983164 g7↔8

84 tgaccaccggggggggcgcctatgcggccg 1989831 1992575 - Rv1759c PE_PGRS(wag22) 914

tgaccaccggggggg-cgcctatgcggccg 1987655 1990398 NOT ANNOTATED AS AN ORF

84 tgaccaccggggggg-cgcctatgcggccg 1983133 1983417 - Mb1790c PE-PGRS FAMILY PROTEIN WAG22A [FIRST PART] 94

MTH:2387369 MTC:2385946 MB:2367769 g3↔2

200 gctcagtggccactcgggcgcccacggcgc 2387200 2387970 - Rv2126c PE_PGRS 256

gctcagtggccactc-ggcgcccacggcgc 2385779 2386845 Non-coding

299 gctcagtggccactcgggcgcccacggcgc 2367600 2368667 - Mb2150c conserved hypothetical protein, PE_PGRS 355

MTH:2614556 MTC:2610404 MB:2592155 g2↔3

acgttgaacaccggt-ggcgatcgggtgcc 2614445 2614654 NOT ANNOTATED AS AN ORF

37 acgttgaacaccggt-ggcgatcgggtgcc 2610292 2610501 + MT2401.2 hypothetical protein 69

acgttgaacaccggtgggcgatcgggtgcc 2592043 2592253 Non-coding

MTH:3462154 MTC:3457952 MB:3418704 cgcgc2↔3

202 cgcggccggccgtgc-----cgcgccgcgc 3461757 3462761 - Rv3093c hypothetical protein Rv3093c 334

cgcggccggccgtgccgcgccgcgccgcgc 3457551 3458560 Non-coding

202 cgcggccggccgtgc-----cgcgccgcgc 3418307 3419311 - Mb3120c HYPOTHETICAL OXIDOREDUCTASE 334

MTH:4197135 MTC:4189453 MB:4133429 a3↔2

acaacctttctgcaa-gccccgggggtcgt 4196549 4197492 Non-coding

119 acaacctttctgcaaagccccgggggtcgt 4188865 4189809 - MT3855 hypothetical protein 314

acaacctttctgcaaagccccgggggtcgt 4132846 4133790 Non-coding

MTH:4369671 MTC:4361975 MB:4306039 g3↔2

165 cccaccgccggccgggccgccgccggtcac 4368515 4370167 - Rv3886c mycP2 membrane anchored serine protease 550

cccaccggcggccgg-ccgccgccggtcac 4360821 4362472 Non-coding

165 cccaccgccggccgggccgccgccggtcac 4304883 4306535 - Mb3916c POSSIBLE SECRETED ALANINE AND PROLINE RICH PROTEASE 550

MTH:1546471 MTC:1546331 MB:1543151 c7↔8

154 gcgaatcgccccccc-gtttgcggaactcg 1546010 1546990 + Rv1373 hypothetical protein Rv1373 326

148 gcgaatcgccccccc-gtttgcggaactcg 1545888 1546850 + MT1418 sulfotransferase, putative 320

154 gcgaatcgccccccccgtttgcggaactcg 1542690 1543487 + Mb1407 GLYCOLIPID SULFOTRANSFERASE [FIRST PART] 265

MTH:1945377 MTC:1936182 MB:1930719 g5↔6

190 atggtgcgccggctgccccccggggcgatc 1944807 1945625 + Rv1718 hypothetical protein Rv1718 272

agatcgccccggggggcagccggcgcacca 1935611 1936429 THIS REGION IS NOT ANNOTATED AS A GENE BUT NEGATIVE STARND(MT1757)

190 agatcgccccggggg-cagccggcgcacca 1930150 1930773 + Mb1746 CONSERVED HYPOTHETICAL PROTEIN 207

MTH:4139181 MTC:4131439 MB:4076531 g7↔8

190 cgggccgcggggggg-tgtgcatgtcaccg 4138199 4139752 - Rv3696c glpK 517

190 cgggccgcggggggg-tgtgcatgtcaccg 4130457 4132010 - MT3798 glycerol kinase 517

191 cgggccgcggggggggtgtgcatgtcaccg 4076348 4077103 - Mb3722c PROBABLE GLYCEROL KINASE GLPKA [FIRST PART] (ATP:GLYCEROL 3-PHOSPHOTRANSFERASE)(GLYCEROKINASE) (GK) 251

MTH:4400658 MTC:4392967 MB:4334622 c2↔3

158 attcgatcgccgacacccgcccggatgctg 4400183 4400851 + 15611047 sigM 222

158 attcgatcgccgaca-ccgcccggatgctg 4392492 4393082 + 15843544 RNA polymerase sigma-70 factor, ECF subfamily 196

158 attcgatcgccgaca-cctcccggatgctg 4334147 4334737 + 31795084 POSSIBLE ALTERNATIVE RNA POLYMERASE SIGMA FACTOR SIGMa [FIRST PART] 196

MTH:125832 MTC:125826 MB:125832 t5↔4

1569 ccggttatcagccac-ttttcgggtgtacc 125641 130539 - Rv0107c ctpI 1632

1569 ccggttatcagccactttttcgggtgtacc 125655 130532 - MT0116 cation-transporting ATPase, E1-E2 family 1625

1569 ccggttatcagccactttttcgggtgtacc 125701 130578 - Mb0111c PROBABLE CATION-TRANSPORTER ATPASE I CTPI 1625

MTH:703174 MTC:704623 MB:704421 g4↔3

121 tgcgatctggtgcgtggggctcacgccgaa 702811 703197 + Rv0607 hypothetical protein 128

126 tgcgatctggtgcgt-gggctcacgccgaa 704245 704688 + MT0636 hypothetical protein 147

121 tgcgatctggtgcgtggggctcacgccgaa 704058 704444 + Mb0623 HYPOTHETICAL PROTEIN 128

MTH:794479 MTC:796455 MB:796250 g3↔2

382 atcctcgttcccaccgggttcatccgcgac 793333 794523 + Rv0694 lldD1 396

382 atcctcgttcccacc-ggttcatccgcgac 795309 796568 + MT0721 FMN-dependent alpha-hydroxy acid dehydrogenase family protein 419

382 atcctcgttcccaccgggttcatccgcgac 795104 796294 + Mb0713 POSSIBLE L-LACTATE DEHYDROGENASE (CYTOCHROME) LLDD1 396

MTH:2858294 MTC:2853785 MB:2825093 c4↔5

143 catgctggtgacccc-gcagctgcgtgcgg 2858252 2858722 - Rv2533c nusB 156

142 catgctggtgacccccgcagctgcgtgcgg 2853340 2854212 - MT2608 N utilization substance protein B 290

143 catgctggtgacccc-gcagctgcgtgcgg 2825051 2825521 - Mb2562c N UTILIZATION SUBSTANCE PROTEIN NUSB (NUSB PROTEIN) 156

MTH:3614510 MTC:3609414 MB:3569115 g3↔2

1030 cgccgcccgcgaggcgggacgcgttgcggc 3614453 3617599 - Rv3239c hypothetical protein Rv3239c 1048

1050 cgccgcccgcgaggc-ggacgcgttgcggc 3609366 3612563 - MT3337 transporter 1065

1029 cgccgcccgcgaggcgggacgcgttgcggc 3569057 3572203 - Mb3267c PROBABLE CONSERVED TRANSMEMBRANE TRANSPORT PROTEIN 1048

MTH:830876 MTC:833068 MB:832700 cg4↔5

8 gacgcggcgcgcgcg--tgaagtggcgctg 830853 831659 + Rv0739 hypothetical protein 268

8 gacgcggcgcgcgcg--tgaagtggcgctg 833045 833851 + MT0764 hypothetical protein 268

21 gacgcggcgcgcgcgcgtgaagtggcgctg 832637 833485 + Mb0760 CONSERVED HYPOTHETICAL PROTEIN 282

MTH:1168715 MTC:1168764 MB:1169166 a3↔2

235 gtctgagagccccgg-aagcgctcgcctag 1168702 1169421 - Rv1046c hypothetical protein 239

235 gtctgagagccccggaaagcgctcgcctag 1168681 1169469 - MT1075.1 hypothetical protein 262

170 gtctgagagccccggaaagcgctcgcctag 1169084 1169677 - Mb1075c HYPOTHETICAL PROTEIN 197

MTH:1994467 MTC:1992290 MB:1985309 c5↔4

439 gatgtttcccctcggccccctccttggcaa 1993151 1994659 + Rv1760 hypothetical protein 502

468 gatgtttcccctcggccccctccttggcaa 1990887 1992482 + MT1809 hypothetical protein 531

439 gatgtttcccctcgg-cccctccttggcaa 1983993 1985522 + Mb1791 CONSERVED HYPOTHETICAL PROTEIN 509

MTH:3037438 MTC:3032377 MB:2994077 gc2↔1

491 accttgcgcgagtttgcgccgcgagcgtaa 3037424 3038911 - Rv2725c hflX 495

491 accttgcgcgagttt--gccgcgagcgtaa 3032179 3033849 - MT2797 GTP-binding protein 556

491 accttgcgcgagtttgcgccgcgagcgtaa 2994063 2995550 - Mb2744c PROBABLE GTP-BINDING PROTEIN HFLX 495

MTH:3531405 MTC:3529042 MB:3488660 t4↔3

79 gcattgtcggggttttgcgccgccgtctac 3531205 3531642 - Rv3162c hypothetical protein 145

79 gcattgtcggggttttgcgccgccgtctac 3528842 3529279 - MT3251 hypothetical protein 145

79 gcattgtcggggttt-gcgccgccgtctac 3488306 3488896 - Mb3187c POSSIBLE INTEGRAL MEMBRANE PROTEIN 196

MTH:4373845 MTC:4366150 MB:4307807 c3↔2

55 gggtttttcgacgcccaggcgcagatgctg 4373723 4374010 - Rv3890c hypothetical protein 95

55 gggtttttcgacgcccaggcgcagatgctg 4366028 4366315 - MT4005 hypothetical protein 95

55 gggtttttcgacgcc-aggcgcagatgctg 4307597 4307971 - Mb3919c PUTATIVE ESAT-6 LIKE PROTEIN 11 124

MTH:1389021 MTC:1388511 MB:1390267 g3↔2

-15 cgatgttgccgcagggcgcttcgtgagcaa 1388683 1388976 - Rv1246c hypothetical protein 97

31 cgatgttgccgcagg-cgcttcgtgagcaa 1388173 1388604 - MT1284 hypothetical protein 143

-15 cgatgttgccgcagggcgcttcgtgagcaa 1389929 1390222 - Mb1278c CONSERVED HYPOTHETICAL PROTEIN 97

MTH:1915541 MTC:1906345 MB:1900883 ac5↔6

5 tgacggcacacacacac--gacgggacacg 1915525 1915908 + Rv1690 lprJ 127

5 tgacggcacacacacac--gacgggacacg 1906329 1906712 + MT1729 hypothetical protein 127

17 tgacggcacacacacacacgacgggacacg 1900833 1901252 + Mb1716 PROBABLE LIPOPROTEIN LPRJ 139

MTH:4134710 MTC:4126969 MB:4072060 g5↔4

-4 cacccgcttgcgggggagagcggcgcagat 4134723 4136045 + Rv3693 hypothetical protein 440

31 cacccgcttgcgggg-agagcggcgcagat 4126876 4128303 + MT3795 conserved hypothetical protein 475

-4 cacccgcttgcgggggagagcggcgcagat 4072073 4073395 + Mb3718 POSSIBLE CONSERVED MEMBRANE PROTEIN 440

MTH:1010205 MTC:1010219 MB:1010672 g2↔3

24 tgctgacgttaacgg-ttgcggatccacgc 1010134 1011732 + Rv0907 hypothetical protein 532

53 tgctgacgttaacgggttgcggatccacgc 1010059 1011747 + MT0930 penicillin-binding protein 4 562

7 tgctgacgttaacgggttgcggatccacgc 1010650 1012200 + Mb0931 CONSERVED HYPOTHETICAL PROTEIN 516

MTH:962954 MTC:962960 MB:963657 g6↔5

22 caggatgagcggggggcagcgcacatggtc 962888 963391 + Rv0864 moaC2 167

22 caggatgagcggggggcagcgcacatggtc 962894 963397 + MT0887 molybdopterin cofactor biosynthesis protein C 167

-3 caggatgagcggggg-cagcgcacatggtc 963665 964093 + Mb0888 PUTATIVE MOLYBDENUM COFACTOR BIOSYNTHESIS PROTEIN C 2 MOAC2 142

MTH:1230417 MTC:1229966 MB:1231789 t3↔2

43 ccgtcggcgcctatttgttcacgccacttt 1229389 1230546 - Rv1101c hypothetical protein 385

43 ccgtcggcgcctatttgttcacgccacttt 1228938 1230095 - MT1133 membrane protein, putative 385

1 ccgtcggcgcctatt-gttcacgccacttt 1230763 1231791 - Mb1131c CONSERVED MEMBRANE PROTEIN 342

MTH:2871994 MTC:2868141 MB:2839447 a6↔5

6 cgaaggtcccaaaaaagccggcgtgcttgg 2871204 2872013 - Rv2552c aroE 269

6 cgaaggtcccaaaaaagccggcgtgcttgg 2867351 2868160 - MT2629 shikimate 5-dehydrogenase 269

-2 cgaaggtcccaaaaa-gccggcgtgcttgg 2838659 2839441 - Mb2582c PROBABLE SHIKIMATE 5-DEHYDROGENASE AROE (5-DEHYDROSHIKIMATE REDUCTASE) 260

MTH:3044892 MTC:3039830 MB:3001531 g2↔3

31 cgaaatcgagccagg-cgcgagggctttgg 3044372 3044986 - Rv2732c hypothetical protein 204

7 cgaaatcgagccagggcgcgagggctttgg 3039310 3039852 - MT2802.1 hypothetical protein 180

31 cgaaatcgagccagg-cgcgagggctttgg 3001011 3001625 - Mb2751c PROBABLE CONSERVED TRANSMEMBRANE PROTEIN 204

MTH:4368178 MTC:4360483 MB:4304546 g3↔2

113 gactacggtcaccgggtcggtgaccgtaga 4366905 4368518 - Rv3885c hypothetical protein Rv3885c 537

-1 gactacggtcaccgg-tcggtgaccgtaga 4359212 4360480 - MT4000 hypothetical protein 422

113 gactacggtcaccgggtcggtgaccgtaga 4303273 4304886 - Mb3915c POSSIBLE CONSERVED MEMBRANE PROTEIN 537

MTH:13235 MTC:13235 MB:13233 g6↔5

108 atcgaaacggtcagcggggggctgctgatt 13133 13558 - Rv0010c hypothetical protein 141

108 atcgaaacggtcagcggggggctgctgatt 13133 13558 - MT0013 hypothetical protein 141

108 atcgaaacggtcagc-gggggctgctgatt 13222 13557 - Mb0010c PROBABLE CONSERVED MEMBRANE PROTEIN 111

MTH:29483 MTC:29465 MB:29463 a3↔2

79 gagcgcgtgagtttcaaacgttcctggtcg 29245 29607 + Rv0025 hypothetical protein 120

79 gagcgcgtgagtttc-aacgttcctggtcg 29227 29499 + MT0028 hypothetical protein 90

79 gagcgcgtgagtttcaaacgttcctggtcg 29225 29587 + Mb0026 CONSERVED HYPOTHETICAL PROTEIN 120

MTH:28935 MTC:28917 MB:28916 c3↔2

191 tggggcgcaacggggcccaaccagttcgac 28362 29207 + Rv0024 hypothetical protein 281

191 tggggcgcaacggggcccaaccagttcgac 28344 29189 + MT0027 NLP/P60 family protein 281

191 tggggcgcaacgggg-ccaaccagttcgac 28343 29176 + Mb0024 PUTATIVE SECRETED PROTEIN P60-RELATED PROTEIN [FIRST PART] 277

MTH:691886 MTC:693336 MB:693132 c7↔8

462 gtggtccggatattg-cccccccgccgccg 690499 692025 + Rv0592 hypothetical protein 508

462 gtggtccggatattg-cccccccgccgccg 691949 693475 + MT0622 virulence factor mce family protein 508

462 gtggtccggatattgccccccccgccgccg 691745 693181 + Mb0607 MCE-FAMILY PROTEIN MCE2DA [FIRST PART] 478

MTH:976896 MTC:976910 MB:977362 a8↔7

435 ctggtgcccccccccaaaaaaaagtaggga 976870 978201 - Rv0878c PPE 443

436 ctggtgcccccccccaaaaaaaagtaggga 976884 978218 - MT0901 PPE family protein 444

435 ctggtgccccccccc-aaaaaaagtaggga 977352 978668 - Mb0902c PPE FAMILY PROTEIN 438

MTH:1299303 MTC:1298793 MB:1300677 c5↔6

166 accgttctcgccccc-ggcgccgatcgcca 1298762 1299802 - Rv1168c PPE 346

166 accgttctcgccccc-ggcgccgatcgcca 1298252 1299292 - MT1205 PPE family protein 346

167 accgttctcgccccccggcgccgatcgcca 1300635 1301177 - Mb1201c PPE FAMILY PROTEIN [FIRST PART] 180

MTH:1468104 MTC:1467647 MB:1466155 cg5↔4

139 agtcccggccgtcgccgcgcgcgcgccgcc 1467686 1468129 + Rv1312 hypothetical protein Rv1312 147

139 agtcccggccgtcgccgcgcgcgcgccgcc 1467229 1467672 + MT1352 conserved hypothetical protein 147

139 agtcccggccgtcgc--cgcgcgcgccgcc 1465737 1466171 + Mb1344 CONSERVED HYPOTHETICAL SECRETED PROTEIN 144

MTH:1951626 MTC:1942428 MB:1936968 g4↔3

41 ccgaaatcgagcggggcctgcccggcatct 1951039 1951749 - Rv1725c hypothetical protein 236

-7 ccgaaatcgagcggg-cctgcccggcatct 1941843 1942406 - MT1766 hypothetical protein 187

41 ccgaaatcgagcggggcctgcccggcatct 1936381 1937091 - Mb1754c CONSERVED HYPOTHETICAL PROTEIN 236

MTH:2563257 MTC:2559105 MB:2540854 tg2↔1

28 tgtcgatcctggatgtgcgctggcggcttg 2563172 2564026 + Rv2291 sseB 284

12 tgtcgatcctggatgtgcgctggcggcttg 2559068 2559874 + MT2348 thiosulfate sulfurtransferase 268

1 tgtcgatcctggatg--cgctggcggcttg 2540851 2541621 + Mb2314 Probable thiosulfate sulfurtransferase SseB 256

MTH:3311132 MTC:3305455 MB:3267823 g2↔3

288 ggttttgaacgccct-ggccgatttacccg 3310711 3311997 - Rv2958c hypothetical protein Rv2958c 428

288 ggttttgaacgccct-ggccgatttacccg 3305034 3306320 - MT3034 UDP-glucoronosyl and UDP-glucosyltransferases family protein 428

289 ggttttgaacgccctgggccgatttacccg 3267589 3268689 - Mb2982c POSSIBLE GLYCOSYL TRANSFERASE [FIRST PART] 366

MTH:3791128 MTC:3783365 MB:3744772 g3↔2

407 tgctcagatgcgattgggtggattcttaat 3790844 3792349 - Rv3377c hypothetical protein Rv3377c 501

407 tgctcagatgcgattgggtggattcttaat 3783081 3784586 - MT3487 cyclase, putative 501

407 tgctcagatgcggtt-ggtggattcttaat 3744541 3745992 - Mb3411c POSSIBLE CYCLASE 483

MTH:4310741 MTC:4303067 MB:4247017 g2↔3

114 tggaccaactgggct-ggcgtgacgaacca 4310398 4310811 + Rv3836 hypothetical protein 137

110 tggaccaactgggct-ggcgtgacgaacca 4302736 4303137 + MT3944 conserved hypothetical protein 133

114 tggaccaactgggctgggcgtgacgaacca 4246674 4247024 + Mb3866 CONSERVED HYPOTHETICAL PROTEIN 116

MTH:935437 MTC:935339 MB:936188 cggccc1↔2

240 gtggtggcatttcga------cggccccgg 934718 935488 + Rv0838 lpqR 256

215 gtggtggcatttcga------cggccccgg 934695 935390 + MT0860 D-alanyl-D-alanine dipeptidase 231

240 gtggtggcatttcgacggccccggccccgg 935469 936245 + Mb0861 PROBABLE CONSERVED LIPOPROTEIN LPQR 258

MTH:968976 MTC:968992 MB:969446 ggc5↔4

422 tagcggtgcccccggcggcggcggcggcgc 968422 970242 - Rv0872c PE_PGRS 606

422 tagcggtgcccccggcggcggcggcggcgc 968428 970257 - MT0894 PE_PGRS family protein 609

421 tagcggtgccccc---ggcggcggcggcgc 968884 970710 - Mb0896c PE-PGRS FAMILY PROTEIN 608

MTH:2639032 MTC:2636366 MB:2607105 gcg5↔4

167 tcggcggcggcggcggcgttggcgccatgg 2637686 2639533 - Rv2356c PPE 615

167 tcggcggcggcggcggcgttggcgccatgg 2635019 2636866 - MT2425 PPE family protein 615

167 tcggcggcggcggcg---ttggcgccatgg 2605762 2607606 - Mb2377c PPE FAMILY PROTEIN 614

MTH:2693078 MTC:2690412 MB:2661153 gcc4↔3

94 gccgaggccgccgccgcctcgcctctgca 2692797 2693882 + Rv2396 PE_PGRS 361

115 gccgaggccgccgccgcctcgcctctgca 2690067 2691215 + MT2467.1 PE_PGRS family protein 382

94 gccgaggccgccgcc---tcgcccctgca 2660871 2661953 + Mb2418 PE-PGRS FAMILY PROTEIN 360

MTH:1612285 MTC:1612143 MB:1608594 tcgacg1↔2

11 agagcgcgtcgacg------gcgcgtacg 1612254 1612391 + Rv1434 hypothetical protein 45

11 agagcgcgtcgacgtcgacggcgcgtacg 1612112 1612255 + MT1478 hypothetical protein 47

11 agagcgcgtcgacg------gcgcgtacg 1608563 1608700 + Mb1469 HYPOTHETICAL PROTEIN 45

MTH:2338200 MTC:2340535 MB:2320092 g8↔11

101 tgtcctgtggtgggt---ggggggggtgtt 2338063 2338503 - Rv2081c hypothetical protein 146

105 tgtcctgtggtgggt---ggggggggtgtt 2340398 2340850 - MT2143 hypothetical protein 150

101 tgtcctgtggtgggtgggggggggggtgtt 2319951 2320394 - Mb2107c POSSIBLE TRANSMEMBRANE PROTEIN 147

MTH:2780761 MTC:2777609 MB:2748924 ggc4↔3

500 cggcgcggcggcggcggctggctccgtcgg 2777386 2782260 - Rv2476c hypothetical protein 1624

500 cggcgcggcggcggcggctggctccgtcgg 2774234 2779108 - MT2551 conserved hypothetical protein 1624

499 cggcgcggcggcggc---tggctccgtcgg 2745551 2750422 - Mb2503c PROBABLE NAD-DEPENDENT GLUTAMATE DEHYDROGENASE GDH (NAD-GDH) (NAD-DEPENDENT GLUTAMIC DEHYDROGENASE) 1623

MTH:2947976 MTC:2944122 MB:2915442 g6↔3

193 gagccttgcaaacatggggggcgccagtca 2947882 2948556 - Rv2621c hypothetical protein 224

193 gagccttgcaaacatggggggcgccagtca 2944028 2944702 - MT2696 hypothetical protein 224

193 gagccttgcaaacat---gggcgccagtca 2915350 2916021 - Mb2654c POSSIBLE TRANSCRIPTIONAL REGULATORY PROTEIN 223

MTH:3528971 MTC:3524459 MB:3484081 gcg5↔4

63 ggtgcggcggcggcggcgatggccgcggcg 3527388 3529160 - Rv3159c PPE 590

76 ggtgcggcggcggcggcgatggccgcggcg 3522876 3524687 - MT3247 PPE family protein 603

63 ggtgcggcggcggcg---atggccgcggcg 3482500 3484269 - Mb3183c PPE FAMILY PROTEIN 589

MTH:3107865 MTC:3102589 MB:3064419 tgg4↔5

33 gcagcggtggtggtggtgg---gcacggtg 3107765 3108394 + Rv2799 hypothetical protein 209

34 gcagcggtggtggtggtgg---gcacggtg 3102487 3103116 + MT2867.1 hypothetical protein 209

34 gcagcggtggtggtggtggtgggcacggtg 3064317 3064949 + Mb2822 PROBABLE MEMBRANE PROTEIN 210

MTH:3583119 MTC:3578026 MB:3537664 ccg4↔3

196 cccgagccgccgccgccgggcatggtgccg 3582529 3583707 - Rv3206c moeZ 392

196 cccgagccgccgccgccgggcatggtgccg 3577436 3578614 - MT3301 HesA/MoeB/ThiF family protein 392

196 cccgagccgccgccg---ggcatggtgccg 3537076 3538251 - Mb3231c PROBABLE MOLYBDENUM COFACTOR BIOSYNTHESIS PROTEIN MOEB1 (MPT-SYNTHASE SULFURYLASE) (MOLYBDOPTERIN SYNTHASE SULPHURYLASE) 391
